# Effects of Chinese medicine aromatherapy combined with positive thought stress reduction training in advanced cancer patients: An unrandomized quasi-experimental trial

**DOI:** 10.1097/MD.0000000000046492

**Published:** 2025-12-12

**Authors:** Shaoju Xie, Qiao Li, Yiyun Li, Xiaoli Zhong, Jiquan Zhang, Fan Xu

**Affiliations:** a Oncology Department, Deyang People’s Hospital, Deyang, Sichuan Province, China; b Nursing Department, Deyang People’s Hospital, Deyang, Sichuan Province, China; c Nephrology Department, Deyang People’s Hospital, Deyang, Sichuan Province, China.

**Keywords:** advanced cancer, Chinese medicine aromatherapy, pain, positive thought stress reduction training, quality of life, sleep quality

## Abstract

**Background:**

Pain and sleep disorders are highly prevalent in advanced cancer patients, seriously affecting their quality of life. At present, the application value of Chinese medicine aromatherapy and positive thought stress reduction training in improving patients’ negative emotions and sleep disorders has been confirmed; however, its intervention role for advanced cancer patients has not yet been widely studied in China. This study investigated the effects of Chinese medicine aromatherapy combined with positive stress reduction training on pain conditions, sleep quality, and quality of life in advanced cancer patients.

**Methods:**

This study is a quasi-experimental study, which included advanced cancer patients admitted to the oncology department of our hospital from July 2022 to December 2022 as the study subjects; the patients were categorized into control and experimental groups according to their wards, with the first ward as the control group and the second ward as the experimental group; the control group implemented the routine nursing care in the oncology department, and the experimental group implemented Chinese medicine aromatherapy combined with positive stress reduction training on the basis of the routine nursing care. McGill Pain Questionnaire Short-Form, Pittsburgh Sleep Quality Index, and Functional Assessment of Cancer Therapy were used to assess the pain, sleep quality, and quality of life before and after the intervention in the 2 groups, and were compared.

**Results:**

A total of 83 patients with advanced cancer aged (59.60 ± 11.29) years completed the study. After the intervention, McGill Pain Questionnaire Short-Form and Pittsburgh Sleep Quality Index scores were lower in the experimental group than in the control group (*P* < .05), and Functional Assessment of Cancer Therapy scores were higher in the experimental group than in the control group (*P* < .05).

**Conclusion:**

Chinese medicine aromatherapy combined with positive stress reduction training can alleviate the pain and sleep condition of advanced cancer patients and improve their quality of life, which is worth promoting.

## 1. Introduction

Cancer is a localized mass formed when the organism is subjected to various tumorigenic factors leading to abnormal proliferation of local tissue cells. As the symptoms of malignant tumors are not obvious and typical in the early stage, the diagnosis is often delayed due to the lack of timely diagnosis, resulting in some patients being in the advanced stage of cancer at the time of diagnosis. Pain and insomnia are common accompanying symptoms of cancer patients, which not only reduce the quality of life of patients but also cause psychological problems and mental symptoms, which bring great pain to patients and families. Studies have found that the prevalence of pain in patients with advanced cancer is as high as 60% to 80%,^[1]^ and the prevalence of their sleep disorders is about 51%.^[2]^

At present, clinical treatment of pain and insomnia in advanced cancer patients is mainly based on drug therapy. However, drug therapy inevitably has side effects and addiction, so it is of practical significance to seek safe and effective non-pharmacological treatments to alleviate the symptoms of pain and insomnia in advanced cancer patients, so as to improve the quality of life of the patients under the premise of effectively alleviating the pain and guaranteeing the safety of the patients.

Aromatherapy in Chinese medicine refers to the use of the aromatic odor of Chinese herbs or their extracted aromatic essential oils, which act on the human body in various forms to regulate the qi of the internal organs and harmonize the yin and yang of the internal organs.^[3]^ Positive thought stress reduction is a kind of intervention method based on meditation, yoga, and body awareness and other positive thought interventions, which awakens the inner focus of the human body, helps patients to carry out individual stress reduction, strengthens the patients’ emotional management ability, and improves their physical and mental regulation ability.^[4]^ Studies have confirmed that Chinese medicine aromatherapy and positive stress reduction can effectively relieve pain and improve sleep quality, thus promoting the physical and mental health of patients.^[5–8]^ In view of this, this study tries to apply the combination of the 2 in the management of pain and sleep and explores the non-pharmacological therapies to relieve pain and improve sleep quality in advanced cancer patients, so as to provide a reference for improving the quality of life of patients.

## 2. Materials and methods

### 2.1. General information

This study is a quasi-experimental study, which included advanced cancer patients admitted to the oncology department of our hospital from July 2022 to December 2022 as the study subjects; the coin toss method was used to group the 2 wards of the oncology department, with the first ward as the control group and the second ward as the experimental group. Inclusion criteria are as follows: histologically or pathologically confirmed diagnosis of malignant tumors, TNM stage Ⅳ; age ≥ 18 years old; clear consciousness, with a certain degree of comprehension; and know the condition and participate voluntarily. Exclusion criteria are as follows: history of allergy, coagulation disorders; the presence of mental illness and cognitive impairment; and combined with severe cardiovascular disease, vital organ failure, or other serious complications.

The sample size was calculated according to the formula for comparing the means of 2 samples: $n=\frac{Z_{\alpha }+Z_{\beta }{}^{2}\times 2\sigma^{2}{{{}^{\;}}^{\;}}_{\;\;}}{\delta^{2}}$, taking α = 0.05, $Z_{\alpha }$ =1.96, β = 0.2, $Z_{\beta }$ =0.84, and using the patients’ pain condition as the main outcome indicator, it is known from reviewing the literature^[9]^ that δ = 1.38, σ=2.95, and substituting it into the formula, n ≈ 39 was calculated, and considering the 10% sample attrition rate, it was determined that the sample size of each group was 44 cases, with a total of 88 cases. The patients were categorized into control and experimental groups according to their wards, with the first ward as the control group and the second ward as the experimental group. The 2 wards, apart from being on different floors, share identical layouts, staffing, and core equipment. Pre-intervention comparisons of patient baseline characteristics, coupled with statistical analyses (*P *> .05), robustly verified group comparability.

### 2.2. Intervention methods

#### 2.2.1. control groups

Implement oncology routine care includes general care, condition observation, drug administration care, nutritional management, and psychological care. The specific content of pain management includes, using visual analog scale (VAS) to assess the patient’s pain situation within 24 hours of admission, according to the principle of 3-step analgesia, timely pain treatment; for patients with pain, the establishment of a pain care record sheet, dynamic assessment of the patient’s pain situation; actively preventing and dealing with adverse reactions after the use of medication; and doing a good job in the patient’s health guidance, which includes Pain assessment methods, measures to relieve pain, principles of drug pain relief, etc. The specific content of sleep management included sleep instruction for patients within 24 hours of admission and on the 3rd day, distribution of paper teaching manuals, playing teaching videos, and instructing patients and their families to fill out sleep diaries. After the patients were discharged from the hospital, the nurse-in-charge made telephone follow-up visits to the patients, which included the patients’ nutritional status, pain control, sleep symptoms, complications, and so on.

#### 2.2.2. Experimental group

On the basis of routine nursing care, Chinese medicine aromatherapy combined with positive thought stress reduction training was implemented. The specific interventions were as follows:

1. Establishment of an intervention team, led by the Department of Oncology, combined with the Department of Traditional Chinese Medicine and the Department of Psychosomatic Medicine, consisting of 2 nurse leaders, 3 doctors, 6 nurses in the Department of Oncology, 2 doctors in the Department of Traditional Chinese Medicine, and 2 psychologists.
2. Members of the division of labor: the head nurse is responsible for coordinating the division of labor, control the progress and quality of the study, the doctor is responsible for answering the patient’s questions about the treatment, the nurse is responsible for the implementation of the specific intervention program, data collection, and statistical analysis, the doctor of traditional Chinese medicine is responsible for the development of the specific implementation of the program of aromatherapy and training, and the psychiatrist is responsible for the implementation of the positive thought stress reduction training.
3. Specific operation of the intervention program:
  a) Explain the purpose and specific content of this study to patients and their families within 24 hours of admission to the hospital, obtain the patients’ informed consent, distribute the publicity materials of Chinese medicine aromatherapy and positive thought stress reduction training, and explain the specific content, operation methods and precautions of Chinese medicine aromatherapy, and positive thought stress reduction training by playing the video combined with the on-site demonstration.
  b) Lavender of the same origin purchased in a unified way is made into a 5 cm × 8 cm specification scented bag of 8~10g per person per time, which is placed in the patient’s pillowcase; the assigned nurse verifies the presence and scent of the sachet daily during rounds, replacing it every 3 days.
  c) 15 minutes before the positive thought stress reduction training, the nurse-in-charge of the training room will put 15-mL lavender essential oil + 15-mL distilled water into the aromatherapy lamp holder, turn on the power supply, so that the smell of lavender essential oil fills the room, the patient is in place, the lavender oil will be applied to the patient’s forehead and behind the ears, to alleviate the patient’s nervousness and anxiety; in the lead of a professional psychotherapist, the patient will be guided to carry out the positive thought stress reduction training, including breathing training, body scanning, positive thought meditation, positive thought raisin, and other 7 topics, at least 1 time a day, 45 minutes each time; confirm that the patient or family members learn the positive thought training intervention methods during hospitalization; and the assigned nurse employs a Mindfulness-Training Checklist to document patients’ attendance and exercise-completion rates, aggregates data daily, and engages poorly adherent patients (e.g., absentees, those with low exercise - completion rates) in discussions to identify reasons and offer tailored guidance. Additionally, the research team conducts a weekly aggregate analysis of sign-in data to detect common adherence-related issues and improves patient compliance by adjusting training schedules, providing supplementary materials and enhancing communication with patients’ families.
  d) After discharge, the made aromatherapy packs were distributed to patients and their families; a WeChat group was set up to send daily videos of the operation of Chinese medicine aromatherapy and positive thought training, and patients and their families were reminded to upload photos or videos of the intervention in the group for punching the clock, and those who had not completed the work as of 20:00 at night were contacted by phone or WeChat to find out the reasons for the lack of adherence and to solve the problems in a timely manner.
  e) The positive stress reduction group training program is shown in Table 1, with 1 session every 7 days for a total of 3 sessions, and the intervention lasted for 21 days.

**Table 1:** Positive thought stress reduction training program.

Time	Topic	Intervention content	Target
Day 1	Breathing exercises	Introduction to the connotation and requirements of breathing training, 30~40min breathing training exercises.	Help patients become clearly aware of their breathing process, improving personal awareness and concentration.
Day 2	Body scanning	Introduction to the connotation and requirements of body scanning, 30~40 min body scanning exercises.	Cultivate “awakening” rather than “falling asleep,” allowing the patient to focus on the whole body, thus achieving a state of deep relaxation.
Day 3	Positive mindfulness meditation	Introduce the connotation and requirements of positive mindfulness meditation, 30~40 min seated meditation practice.	Enables the patient to focus on the whole body, thus achieving a state of deep relaxation.
Day 4	Positive thought raisin	Introducing the connotation and requirements of positive thought raisin, 30~40min positive thought raisin practice.	Enhance the patient’s awareness and appreciation of the present moment.
Day 5	Positive thought walking	Introduce the connotation and requirements of positive thought walking, 30~40min positive thought walking practice.	Help patients feel the earth against the soles of their feet to increase their strength.
Day 6	Positive lying	Introducing the connotation and requirements of Positive Lying Pose, 30~40 min Positive Lying practice.	Alleviating the patient’s bad mood and relaxing the body and mind.
Day 7	Loving-kindness meditation	Introduction to the content and requirements of loving-kindness meditation, 30~40 min loving-kindness meditation practice.	Realizing self-healing.

### 2.3. Evaluation instruments

#### 2.3.1. General information questionnaire

It was self-administered by the researcher and included age, gender, education, marital status, occupation, disease duration, and cancer type.

#### 2.3.2. McGill Pain Questionnaire Short-Form

The questionnaire was compiled by Melzack,^[10]^ including 3 subscales: Pain Rating Index (PRI), Pain VAS, and present pain intensity, and the total score was the sum of the 3 subscales; PRI: a total of 11 sensory items, and the pain level was scored 0 to 3, with a total score of 0 to 33, and the higher the score, the more obvious the pain. VAS: score range 0~10, with 0 indicating no pain and 10 indicating severe pain; present pain intensity: score range 0~5, with higher scores indicating more obvious pain.

#### 2.3.3. Pittsburgh Sleep Quality Index

Compiled by Buysse in 1989^[11]^ and Chineseized by Liu Xianchen in 1996,^[12]^ it is used to assess the sleep quality of an individual, the scale contains 7 dimensions of sleep quality, time to fall asleep, sleep duration, sleep efficiency, sleep disorders, and hypnotic medication, with a total of 24 entries, and the dimensions are scored on a scale of 0 to 3, with a total score of 0 to 21, and a total score of ≥8 indicates poor quality of sleep, and the scale Cronbach α coefficient was 0.842.

#### 2.3.4. Functional assessment of cancer

The scale was developed by Cella^[13]^ and translated and Chineseized by Wan Chonghua^[14]^ to assess the quality of survival of cancer patients. The scale consists of 27 entries in 4 dimensions, namely physiological condition, social/family condition, emotional condition, and functional condition, and is based on a 5-point Likert scale, where the scores of the dimensions are summed by the scores of the corresponding entries, and the total score is summed by the scores of the dimensions, and the range of the total score is from 0 to 108, and the higher the score is, the better the quality of life is. Lili Zhang tested the scale in Chinese cancer patients and showed that the Cronbach alpha coefficient for each domain of the scale was 0.907.^[15]^

### 2.4. Data collection

Before the intervention, 1 nurse who was not involved in the intervention and had received homogenization training distributed the questionnaire within 24 hours of the patient’s admission; 21 days after the intervention, the same nurse distributed the questionnaire for the survey. To ensure the standardization and consistency of the intervention program, a unified operation manual, technical specifications, contingency plan, and training plan were formulated before the start of the project, and Chinese medicine doctors and psychological counselors were invited to provide unified training to those who participated in the study.

### 2.5. Statistical analyze

SPSS26.0 software was used for statistical analysis, and the measurement data were described by mean ± standard deviation, and the counting data were described by number of cases and constitutive ratio; the comparison of baseline data between the 2 groups was analyzed by 2 independent samples *t*-test and chi-square test, and the comparison of the evaluation indexes before and after the intervention of the 2 groups was analyzed by paired samples *t*-test, and the difference was statistically significant at *P* < .05. In cases of non-withdrawal-related missing data for some patients, the multiple imputation method was applied to handle such data.

## 3. Results

### 3.1. Comparison of general information between the 2 groups

**Figure 1.**
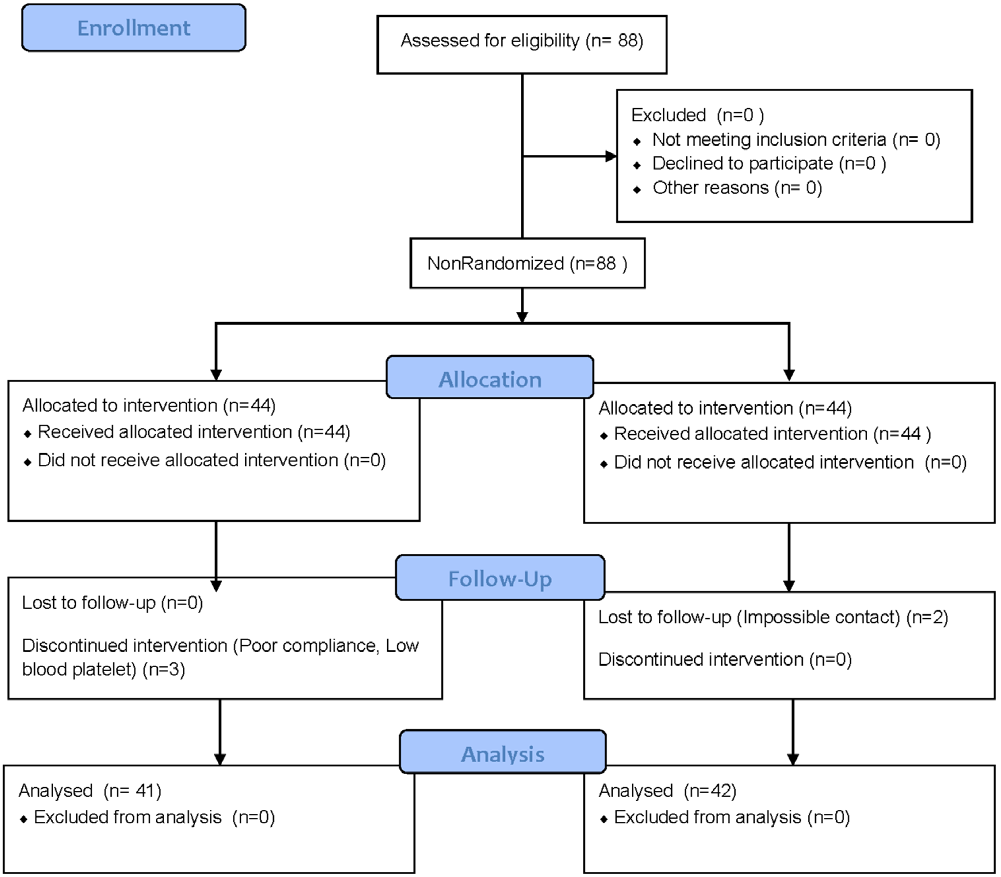
Study flowchart.

In this study, a total of 83 advanced cancer patients completed the study, aged (59.60 ± 11.29) years, 2 patients in the experimental group did not complete the study due to poor compliance, 1 patient was not suitable for continued intervention due to low platelets (Fig. 1); 2 patients in the control group did not complete the study because they could not be contacted after discharge from the hospital, totaling 42 cases in the control group and 41 cases in the experimental group. Comparing the general information of the 2 groups, the difference was not statistically significant (*P > *.05), and the distribution between the groups was balanced (Table 2 and Fig. 1).

**Table 2:** Comparison of general information between the 2 groups.

Variables		Control group (n = 42)	Experimental group (n = 41)	t/χ^2^	*P*
Age (yr, mean ± SD)		59.02 ± 11.38	60.20 ± 11.29	−0.471	.639*
Sex	Male	28	26	0.097	.756†
	Female	14	15		
Education level	Primary and below	16	15	1.070	.784†
	Junior high school	12	14		
	High school/Junior college	7	8		
	College and above	7	4		
Marital status	Married	28	25	0.291	.589†
	Unmarried/Divorced/Widowed	14	16		
Careers	Incumbency	8	11	3.039	.386†
	Retirement	9	11		
	Unemployed	14	7		
	Other	11	12		
Disease duration	<1 yr	10	3	4.951	.083‡
	1~5 yr	23	30		
	>5 yr	9	8		
Type of cancer	Lung cancer	21	17	5.194	.268‡
	Stomach cancer	5	13		
	Colorectal cancer	5	3		
	Breast cancer	5	3		
	Other	6	5		

SD = standard deviation.

* *t*-test.

† Chi-square test.

‡ Calibrated chi-square test.

### 3.2. Comparison of pain before and after intervention in the 2 groups

**Figure 2.**
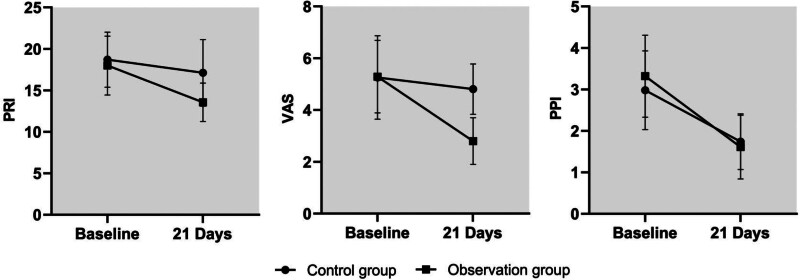
Comparison of SF-MPQ scale and subscale pain scores between the 2 groups. SF-MPQ = McGill Pain Questionnaire Short-Form.

After the intervention, the McGill Pain Questionnaire Short-Form (SF-MPQ) and the scores of PRI and VAS subscales were lower in the experimental group than in the control group (*P < *.05). See Table 3 and Figure 2.

**Table 3:** Comparison of SF-MPQ scale and subscale pain scores between the 2 groups.

Variables		Control group (n = 42)	Experimental group (n = 41)	*t*	*P*	Cohen *d*	95% CI
PRI, mean ± SD	pre	18.71 ± 3.32	18.00 ± 3.55	0.948	.346	0.208	−0.224 to 0.639
	post	17.12 ± 4.01	13.56 ± 2.32	4.957	<.001	1.082	0.617 to 1.540
VAS, mean ± SD	pre	5.26 ± 1.61	5.29 ± 1.40	−0.093	.926	−0.020	−0.451 to 0.410
	post	4.81 ± 0.97	2.80 ± 0.90	9.759	<.001	2.142	1.596 to 2.680
PPI, mean ± SD	pre	2.98 ± 0.95	3.32 ± 0.99	−1.604	.112	−0.352	−0.785 to 0.083
	post	1.74 ± 0.67	1.61 ± 0.77	0.813	.419	0.178	−0.253 to 0.609
Total score of SF-MPQ, mean ± SD	pre	26.95 ± 3.99	26.61 ± 3.82	0.399	.691	0.088	−0.343 to 0.518
	post	23.67 ± 4.39	17.98 ± 2.81	7.050	<.001	1.540	1.045 to 2.027

post = after intervention, PPI = present pain intensity, pre = before intervention, PRI = Pain Rating Index, SD = standard deviation, SF-MPQ = McGill Pain Questionnaire Short-Form, VAS = pain visual analog scale.

### 3.3. Comparison of sleep quality between the 2 groups before and after intervention

**Figure 3.**
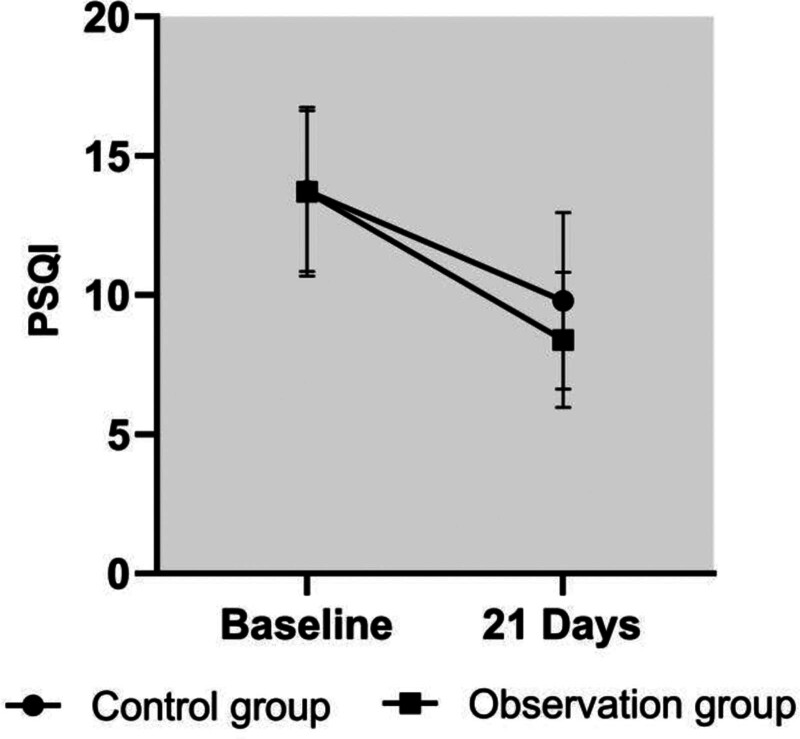
Comparison of PSQI scale scores between the 2 groups. PSQI = Pittsburgh Sleep Quality Index.

After the intervention, Pittsburgh Sleep Quality Index scores were lower in the experimental group than in the control group (*P < *.05). See Table 4 and Figure 3.

**Table 4:** Comparison of PSQI scale scores between the 2 groups.

Variables		Control group (n = 42)	Experimental group (n = 41)	*t*	*P*	Cohen *d*	95% CI
Total score of PSQI, mean ± SD	pre	13.74 ± 2.89	13.71 ± 3.03	0.047	.962	0.010	−0.420 to 0.441
	post	9.79 ± 3.17	8.39 ± 2.43	2.253	.027	0.493	0.055 to 0.929

post = after intervention, pre = before intervention, PSQI = Pittsburgh Sleep Quality Index, SD = standard deviation.

### 3.4. Comparison of quality of life between the 2 groups before and after the intervention

**Figure 4.**
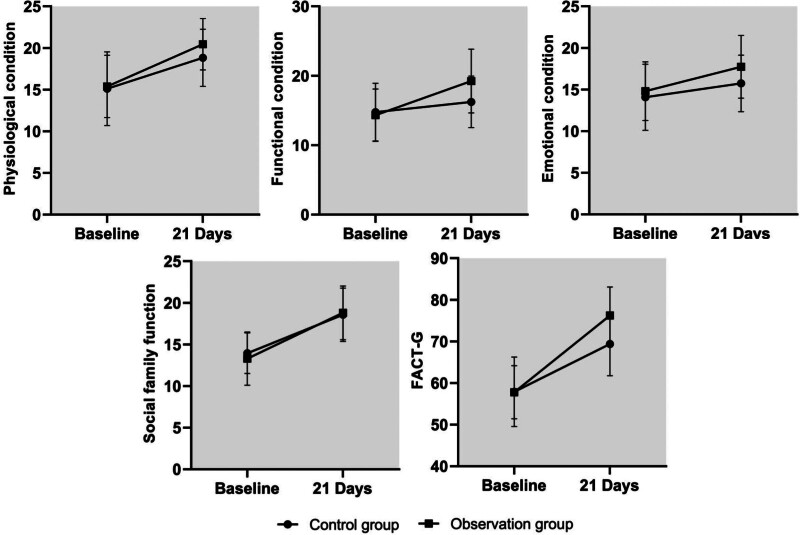
Comparison of FACT-G scale and dimension scores between the 2 groups. FACT-G = Functional Assessment of Cancer Therapy.

After the intervention, SF-MPQ and physiological condition, functional condition, and emotional condition scores were higher in the experimental group than in the control group (*P* < .05). See Table 5 and Figure 4.

**Table 5:** Comparison of FACT-G scale and dimension scores between the 2 groups.

Variables		Control group (n = 42)	Experimental group (n = 41)	*t*	*P*	Cohen *d*	95% CI
Physiological condition, mean ± SD	pre	15.12 ± 4.42	15.39 ± 3.75	-0.301	.764	−0.066	−0.496 to 0.364
	post	18.83 ± 3.43	20.46 ± 3.08	−2.279	.025	−0.496	−0.927 to −0.061
Social family function, mean ± SD	pre	13.95 ± 2.44	13.29 ± 3.20	1.054	.295	−0.073	−0.503 to 0.359
	post	18.57 ± 3.20	18.80 ± 3.23	−0.331	.742	0.232	−0.200 to 0.663
Functional condition, mean ± SD	pre	14.76 ± 4.16	14.32 ± 3.76	0.511	.611	0.112	−0.319 to 0.542
	post	16.24 ± 3.71	19.24 ± 4.59	−3.285	.002	−0.721	−1.163 to −0.275
Emotional condition, mean ± SD	pre	14.07 ± 3.97	14.80 ± 3.52	−0.890	.376	−0.195	−0.626 to 0.237
	post	15.74 ± 3.40	17.73 ± 3.77	−2.532	.013	−0.556	−0.993 to −0.115
Total score of FACT-G, mean ± SD	pre	57.90 ± 8.33	57.80 ± 6.37	0.061	.951	0.013	−0.417 to 0.444
	post	69.38 ± 7.62	76.24 ± 6.84		<.001	−0.947	−1.399 to −0.490

FACT-G = Functional Assessment of Cancer Therapy, post = after intervention, pre = before intervention, SD = standard deviation.

## 4. Discussion

The World Health Organization points out that cancer pain management is an important global challenge. The results of this study showed that after the intervention, the SF-MPQ, PRI, and VAS subscale scores were lower than those of the control group (*P* < .05), indicating that Chinese medicine aromatherapy combined with orthostatic stress reduction training can effectively alleviate the pain of patients with advanced cancer, which is similar to the results of Chao study.^[16]^ Chinese medicine believes that tumor is a Yin-cold syndrome, and the occurrence of cancer pain is related to Yin-cold evil, and cancer pain is categorized as “pain syndrome,” and it is believed that “if it is not honored, it will be painful” and “if it is not communicated, it will be painful,” and the treatment should focus on supporting and tonifying Qi, promoting Qi circulation and dispersing knots. It is believed that “not glorifying is painful” and “not communicating is painful.” Chinese medicine aromatherapy will have the aromatic odor of the agent through inhalation, skin mucous membrane pathway effect on the human body local or whole body, and play the role of opening the coupling, blood circulation, and the role of a wide range of applications in the field of cancer patients’ symptom management, such as sleep, pain, anxiety, depression, etc, it is a clinically commonly used adjunctive therapies, and compared with the traditional drug management, it has the operation of the characteristics of simple and high comfort level. Relevant literature pointed out that the effect of Chinese medicine aromatherapy combined with the 3-step pain relief method to relieve pain is better than a single means.^[17]^ And Positive Thought Stress Reduction Training can help patients gain control of their body and mind through positive thought meditation, body awareness, and perception of the present moment, thus improving their pain beliefs and somatic sensations. It has been pointed out that the intervention based on positive thoughts can intervene in nociceptive afferents and reduce pain through cortically driven neural circuits.^[18]^ Therefore, in this study, based on the conventional 3-step pain relief method, the combination of Chinese medicine aromatherapy and positive thought stress reduction training can effectively relieve pain in advanced cancer patients.

The results of this study showed that after the intervention, the Pittsburgh Sleep Quality Index scores of the experimental group were lower than those of the control group (*P* < .05), indicating that Chinese medicine aromatherapy combined with positive thought stress reduction training can alleviate the sleep of patients with advanced cancer, which is similar to the results of patient.^[19]^ According to Chinese medicine, “Yang energy is awake, and yin energy is asleep,” and sleep is a physiological activity that balances yin and yang. In this study, Chinese medicine aromatherapy was organically combined with positive thought stress reduction training. Compared with the traditional intervention, the main line of “yin and yang balance” is to fully explore the patients’ senses of smell, hearing, taste, and touch, so as to bring better soothing experience to the patients, and the lavender essential oil and scented sachet in Chinese medicine aromatherapy can regulate the sleep-related neurotransmitters and enhance the activity of parasympathetic nerves,^[20,21]^ thus improving the sleep condition of the patients. Positive thought stress reduction training can help patients to face the disease, accept the disease, and feel the present moment with a positive mindset in their life, strengthen the ability of self-emotional management, reject negative concepts and bad emotions, and improve the quality of sleep of the patients through positive thought meditation and raisin exercises. The psychotherapist guides the patients in the process of breathing exercises and becoming aware of their own bodies, which, together with the atmosphere of Chinese medicine aromatherapy, allows the patients to feel the process of physical relaxation, promotes the relaxation of muscle states, and immerses the whole body and mind in a calm, soft, and soothing state of mind, so that the patients can enter into the sleep state faster and easier, thus improving the quality of sleep of the patients.

The results of this study showed that after the intervention, the SF-MPQ and the scores of physiological condition, functional condition, and emotional condition of the experimental group were higher than those of the control group (*P* < .05), which indicated that Chinese medicine aromatherapy combined with positive stress reduction training could effectively improve the quality of life of patients with advanced cancer, which was similar to the results of Rivaz study.^[18]^

From the Yin-Yang theory, the heart and the lungs in the body on the operation of qi and blood influence each other, the heart of the blood, for the monarch of the official, carries the lungs, promoting the transmission of qi, blood, and fluid; lungs toward the hundred veins, the main body of the gas, the division of breathing, the opening of the orifices in the nose, the lungs to help the heart line of blood, and the nose is the main ventilation and the main olfactory sense. In this study, by inhaling and smelling the aromatic components of natural plant essential oils, we stimulate the human olfactory system, and with the transportation of the heart and blood, we realize the effective regulation of the lungs on the transportation of qi to reach the therapeutic effect, so as to improve the quality of life of the patients. For advanced cancer patients, long-term disease torture, mental trauma, economic pressure, and other factors will lead to stress response of the organism and physiological dysfunction, which will lead to a decline in the quality of life, and this study through the combination of Chinese medicine aromatherapy and positive stress reduction, so that the patients in the end stage of the disease pain symptoms have been improved, no longer suffer from pain all the time, and due to the improvement of the sleep disorders also led to the improvement of various functions of the patient’s body, which achieved the purpose of improving the quality of life.^[22,23]^

## 5. Conclusions

Chinese medicine aromatherapy combined with positive thought stress reduction training can effectively relieve the pain condition and improve the sleep quality of advanced cancer patients, and its operation is simple and safe. However, this study has several limitations. First, the sample may be limited due to recruitment solely from the oncology department of Deyang People’s Hospital, and the study’s short duration prevents confirmation of long-term effectiveness. Second, the unblinded study design may have created participant expectation bias, which could compromise the objectivity of outcome assessment. The observed improvements in pain perception, sleep quality, and quality of life in the intervention group may partly result from the additional attention they received rather than the intervention alone. Finally, data collection based on self-reports from unblinded participants may be subject to expectation bias, potentially leading to an overestimation of the results. Therefore, future studies can conduct multicenter studies, select multiple time points, and extend the intervention time for the tracking of the intervention effect, so as to conduct in-depth research on the generalizability and long-term effects of the intervention program. Additionally, implementing blinded study designs, establishing simulated control and placebo groups, and integrating objective measurement indicators would enhance the precision of assessing the intervention’s genuine efficacy. These methodological refinements would help mitigate the placebo effect and expectation bias, ultimately bolstering the study’s scientific rigor and the robustness of its findings.

## Acknowledgments

The authors express their gratitude to all the older adults who participated in the study.

## Author contributions

**Conceptualization:** Shaoju Xie, Qiao Li, Yiyun Li, Xiaoli Zhong, jiquan Zhang, Fan Xu.

**Data curation:** Shaoju Xie, Qiao Li, Yiyun Li, jiquan Zhang, Fan Xu.

**Formal analysis:** Shaoju Xie, Xiaoli Zhong, jiquan Zhang, Fan Xu.

**Investigation:** Shaoju Xie, Qiao Li, Yiyun Li, jiquan Zhang, Fan Xu.

**Methodology:** Shaoju Xie.

**Project administration:** Shaoju Xie.

**Writing – original draft:** Shaoju Xie, Fan Xu.

**Writing – review & editing:** Shaoju Xie, Qiao Li, Yiyun Li, Xiaoli Zhong, jiquan Zhang, Fan Xu.
